# Diversity of IncP-9 plasmids of *Pseudomonas*

**DOI:** 10.1099/mic.0.2008/017939-0

**Published:** 2008-10

**Authors:** Yanina R. Sevastsyanovich, Renata Krasowiak, Lewis E. H. Bingle, Anthony S. Haines, Sergey L. Sokolov, Irina A. Kosheleva, Anastassia A. Leuchuk, Marina A. Titok, Kornelia Smalla, Christopher M. Thomas

**Affiliations:** 1 School of Biosciences, University of Birmingham, Edgbaston, Birmingham B15 2TT, UK; 2 Skryabin Institute of Biochemistry and Physiology of Microorganisms, Russian Academy of Sciences, Pushchino 142290, Russia; 3 Genetics Department, Biology Faculty, Belarus State University, 6 Kurchatova St, Minsk 220064, Belarus; 4 Julius Kühn Institute – Federal Research Centre for Cultivated Plants (JKI), Messeweg 11/12, 38104 Braunschweig, Germany

## Abstract

IncP-9 plasmids are important vehicles for degradation and resistance genes that contribute to the adaptability of *Pseudomonas* species in a variety of natural habitats. The three completely sequenced IncP-9 plasmids, pWW0, pDTG1 and NAH7, show extensive homology in replication, partitioning and transfer loci (an ∼25 kb region) and to a lesser extent in the remaining backbone segments. We used PCR, DNA sequencing, hybridization and phylogenetic analyses to investigate the genetic diversity of 30 IncP-9 plasmids as well as the possibility of recombination between plasmids belonging to this family. Phylogenetic analysis of *rep* and *oriV* sequences revealed nine plasmid subgroups with 7–35 % divergence between them. Only one phenotypic character was normally associated with each subgroup, except for the IncP-9*β* cluster, which included naphthalene- and toluene-degradation plasmids. The PCR and hybridization analysis using pWW0- and pDTG1-specific primers and probes targeting selected backbone loci showed that members of different IncP-9 subgroups have considerable similarity in their overall organization, supporting the existence of a conserved ancestral IncP-9 sequence. The results suggested that some IncP-9 plasmids are the product of recombination between plasmids of different IncP-9 subgroups but demonstrated clearly that insertion of degradative transposons has occurred on multiple occasions, indicating that association of this phenotype with these plasmids is not simply the result of divergent evolution from a single successful ancestral degradative plasmid.

## INTRODUCTION

As agents of horizontal gene transfer (HGT), plasmids contribute considerably to bacterial evolution and adaptation (Thomas, 2000a). Plasmids are extremely diverse and distributed universally in a range of hosts and ecological niches (Turner *et al.*, 2002). Until recently the available molecular tools, such as primers and probes, for identification and classification of plasmids from Gram-negative bacteria originated largely from clinical studies (Couturier *et al.*, 1988; Gotz *et al.*, 1996). The limited usefulness of these in broad ecological studies prompted projects to catalogue and characterize diverse replicons from various environmental contexts (Dahlberg *et al.*, 1997; Kobayashi & Bailey, 1994; Sobecky *et al.*, 1997). Plasmid sequencing also provides valuable insights into their evolution, confirming their mosaic nature based on genetic modules of different origins, and indicating the importance of recombination, cointegration and mobile element insertions [insertion sequence (IS) elements, transposons and integrons] in plasmid diversity (Boyd *et al.*, 1996; Osborn *et al.*, 2000; Tan, 1999; Thomas, 2000b; Toussaint & Merlin, 2002; Tsuda *et al.*, 1999).

Species of the *Pseudomonas* genus are found in most soil and aquatic environments and some are implicated in diseases of humans, animals and plants (Wackett, 2003). Natural isolates of *Pseudomonas* often harbour plasmids, which are currently classified into 14 incompatibility groups (Boronin, 1992; Thomas & Haines, 2004). Plasmid-encoded traits contribute to the genetic plasticity and adaptability of pseudomonads in various ecological niches. The IncP-9 plasmid family includes large self-transmissible plasmids associated with degradation and antibiotic- and toxic metal-resistance markers (Korfhagen *et al.*, 1978). The best-known IncP-9 plasmids are pWW0 (Williams & Murray, 1974) and NAH7 (Dunn & Gunsalus, 1973), the paradigms of prokaryotic degradation of mono- and polyaromatic compounds (toluene/xylenes and naphthalene) (Eaton, 1994; Harayama & Rekik, 1989; Kasai & Harayama, 2004; Ramos *et al.*, 1997; Yen & Gunsalus, 1982), and these have been frequently used in natural and genetically modified biodegraders for bioremediation of polluted sites (Lee *et al.*, 1995; Nüsslein *et al.*, 1992; Panke *et al.*, 1998; Parales *et al.*, 2002; Ramos & Timmis, 1987; Ramos *et al.*, 1987; Top *et al.*, 2002).

Hybridization studies with IncP-9 plasmids R2, pMG18, NAH7, pWW0 and SAL (Bayley *et al.*, 1979; Heinaru *et al.*, 1978; Lehrbach *et al.*, 1983) have indicated that diverse IncP-9 replicons share about 6 kb of DNA sequence that must encode functions essential for plasmid survival (‘core sequence’). When sequences of IncP-9 plasmids pMT2, pWW0, pDTG1 and NAH7 became available the relationships between them were estimated by sequence comparison and phylogenetic analysis (Dennis & Zylstra, 2004; Dennis, 2005; Greated & Thomas, 1999; Greated *et al.*, 2002; Sota *et al.*, 2006). PCR primers that target the basic replicon functions of IncP-9 have facilitated studies on the diversity and prevalence of naturally occurring IncP-9 plasmids (Greated & Thomas, 1999; Krasowiak *et al.*, 2002). Thus, the genetic diversity of a limited number of phenotypically different IncP-9 plasmids (Krasowiak *et al.*, 2002) and large collections of IncP-9 Nah replicons (Izmalkova *et al.*, 2006; Leuchuk *et al.*, 2006) have been evaluated by RFLP analysis of replication/maintenance loci, selected *nah* determinants or complete plasmid genomes. Environmental studies indicate a wide distribution of IncP-9-like replicons in manure, soils and coastal waters and their involvement in natural HGT of the naphthalene-degradation trait (Herrick *et al.*, 1997; Krasowiak *et al.*, 2002; Smalla *et al.*, 2000).

Collaboration with groups in Russia, Belarus and Germany made available a large collection of IncP-9 plasmids, while complete plasmid sequences and tools for analysis and detection of IncP-9 replicons allow analysis of genetic diversity among these plasmids using PCR, sequencing and hybridization. This study presents a subclassification of IncP-9 plasmids based on sequence analysis of the replication loci, *oriV* and *rep*. Subsequent analysis of selected backbone loci indicated that phylogenetically distant IncP-9 plasmids must retain many common genes, while modules encoding similar phenotypes have been inserted at different sites in the conserved plasmid backbone, indicating multiple independent acquisitions of these functions.

## METHODS

### Bacterial strains, plasmids and growth conditions.

*Pseudomonas* strains carrying IncP-9 plasmids used in this study are listed in Table 1. Additional strains used were *Pseudomonas putida* BS 394 (*cys*^−^ Rif^r^ Str^r^) (Russian Academy of Sciences collection) and *Escherichia coli* DH5*α* (F^−^ *φ*80d*lacZ*ΔM15 *endA thi-1 recA1 gyrA96 relA1 hsdR17 supE44 deoR* Δ(lacZYA*-argF*) U169; Gibco-BRL). Bacteria were propagated in Luria broth or M9 minimal medium, supplemented with 1.5 % agar when required (Sambrook & Russell, 2001). The cultivation temperature was 30 °C for *Pseudomonas* spp. and 37 °C for *E. coli*. To select for catabolic plasmids, bacteria were maintained on M9 lacking glucose but supplemented with an appropriate source of carbon and energy such as naphthalene [0.05–0.1 g naphthalene (Sigma) placed on the lid of an inverted Petri dish], caprolactam and toluene (0.1–0.15 % and 5 mM added to molten agar before pouring, respectively). Cysteine and proline (40 μg ml^−1^) were added for growth of auxotrophic strains. Antibiotic-resistance plasmids were retained by supplementing medium with antibiotics as follows: ampicillin, 100 μg ml^−1^; kanamycin, 50 μg ml^−1^; tetracycline, 10–25 μg ml^−1^; streptomycin, 50 μg ml^−1^ and gentamicin, 10 μg ml^−1^. PCR products were cloned into a T-overhang vector (pGEM-T Easy; Promega).

### DNA sequences.

Published plasmid sequences used in this study were: pMT2 (accession no. AF078924), pWW0 (AJ344068), pDTG1 (AF491307), NAH7 (AB237655), pBBR1 (X66730) and pBI709 (partial sequence, AY299015). Newly determined sequences generated in this work were deposited in GenBank Database under the accession numbers EU499619–EU499641 for *oriV* and EU499644–EU499666 for *rep*.

### DNA isolation and manipulation.

Plasmid DNA was extracted for screening purposes using the alkaline lysis method (Birnboim & Doly, 1979) or with a Wizard Plus SV Miniprep kit (Promega) for sequencing. Large plasmids were isolated with a Qiagen Midiprep kit and total genomic DNA was extracted using a GenElute Bacterial Genomic DNA kit (Sigma). Restriction endonuclease digestion and agarose gel electrophoresis were carried out using established techniques (Sambrook & Russell, 2001). Restriction enzymes were from New England Biolabs, MBI Fermentas, Invitrogen and Boehringer Mannheim (Roche). PCR products were purified from agarose gel or reaction mixtures using a High Pure PCR Product Purification kit (Roche) or GeneClean Spin kit (Bio 101). Bacteria were transformed using a standard CaCl_2_ transformation protocol (Sambrook & Russell, 2001).

### PCR amplification of *rep*, *oriV* and selected backbone loci.

PCR primers used are listed in Supplementary Table S1 available with the online version of this paper. Putative *rep* and *oriV* were amplified from IncP-9 plasmids with *rep*F and *rep*R (Greated & Thomas, 1999) and *oris*F and *oris*R primer pairs. PCR was performed using Bio-X-Act DNA Polymerase (Bioline); total genomic DNA or crude cell lysate was used as a DNA template. The reaction included 3 mM MgCl_2_, 0.8 mM dNTPs, 0.2 μM of each primer and 2–4 U polymerase in 1× PCR buffer provided with the polymerase. The thermal cycling profile used was: 5 min denaturation at 94 °C; 30 cycles of denaturation at 94 °C for 1 min, annealing at a specific annealing temperature (*T*_a_) (Supplementary Table S1) for 1 min and elongation at 68 °C for 1 min; final extension at 68 °C for 10 min. Selected backbone loci were amplified from IncP-9 plasmids (total genomic DNA or crude cell lysate) using *Taq* polymerase (Invitrogen) according to the manufacturer's instructions. The cycling profile was as follows: 5 min denaturation at 94 °C; 30 cycles of denaturation at 94 °C for 1 min, annealing at *T*_a_ (Supplementary Table S1) for 1 min, extension at 72 °C for 1 min (or 2.5 min for the last two primer pairs in Supplementary Table S1 due to the increased size of the product) and final extension at 72 °C for 10 min.

### DNA sequencing.

Automated sequencing was carried out using the Dye Terminator Cycle Sequencing Ready Reaction kit (Perkin-Elmer Applied Biosystems) on an ABI Prism 3700 DNA automatic sequencer (Functional Genomics Laboratory, University of Birmingham) according to the manufacturer's instructions. Putative IncP-9 *rep* PCR products (∼500 bp) were cloned in the pGEM-T Easy vector and sequenced on both strands with pUC18/M13 primers. Purified *oriV* PCR products (540–600 bp) were sequenced directly using 30–50 ng template and 6 pmol *oris*F or *oris*R primer.

### Southern blotting.

Selected PCR products or *Sal*I- or *Ava*I-digested total genomic DNA (300–500 ng) from strains with IncP-9 plasmids were fractionated by 1.5 % agarose gel electrophoresis in Tris-acetate/EDTA buffer and blotted to Hybond-N+ membranes (Amersham) by neutral capillary transfer using 20× SSC buffer [0.3 M sodium citrate, 3 M NaCl, pH 7.0 (Sambrook & Russell, 2001)] for 16 h. DNA was fixed by UV-induced cross-linking at 70 mJ cm^−2^. Hybridization probes were generated from relevant PCR products (Supplementary Table S1) by random priming labelling with a DIG DNA Labeling and Detection kit (Roche) following the manufacturer's instructions. Hybridizations were performed overnight in hybridization buffer (5×SSC, 0.1 % *N*-lauroylsarcosine, 0.02 % SDS, 1 % blocking reagent) at 50 °C, followed by two washes in 2×SSC, 0.1 % SDS at room temperature and two washes in 0.1×SSC, 0.1 % SDS at 55 °C. The blots were developed using nitro-blue tetrazolium chloride/5-bromo-4-chloro-3′-indolyphosphate *p*-toluidine salt (NBT/BCIP) colour substrate solution, and the image was acquired on a GS-710 densitometer (Functional Genomics Laboratory, University of Birmingham).

### Computer analysis.

DNA sequence alignments were generated using the pileup programme from the UWGCG package (Devereux *et al.*, 1984) and edited manually. Phylogenetic analysis was performed using the neighbour-joining (Saitou & Nei, 1987) and maximum-parsimony methods as implemented by clustal_x (v1.83) (Thompson *et al.*, 1997), phylip (Felsenstein, 1989) and mega v3.1 (Tamura *et al.*, 2007) program packages. Phylogenetic trees were displayed with TreeView (v1.6.6) (Page, 1996). Alignments of complete plasmid sequences were obtained using the ACT program from the Artemis package (Rutherford *et al.*, 2000). Protein identity values were determined using blast (Altschul *et al.*, 1997).

## RESULTS

### Phylogeny of the IncP-9 plasmid family

**Fig. 1. f1:**
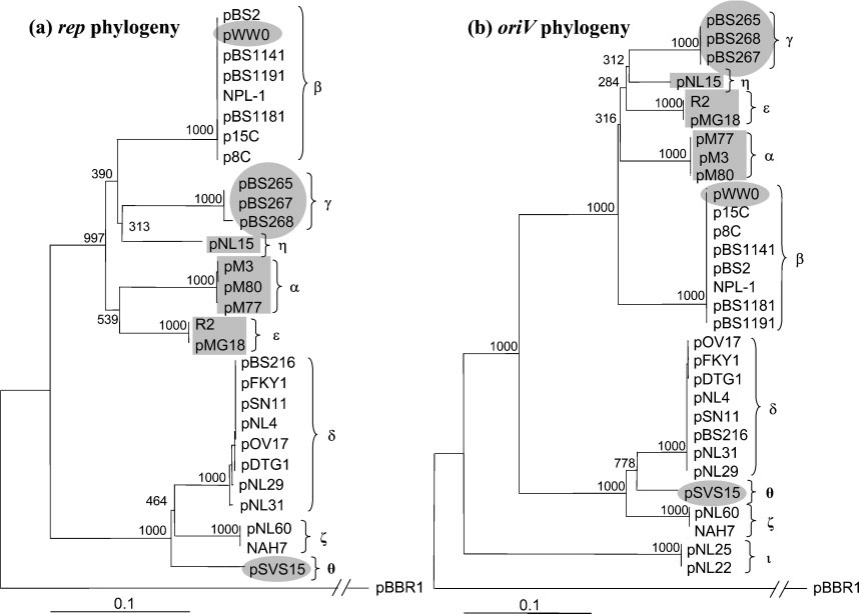
Neighbour-joining rooted phylogenies of the IncP-9 plasmid family based on sequence analysis of *rep* (a) and *oriV* (b) loci. Plasmid subgroups are bracketed and named with letters of the Greek alphabet from *α* to ι; grey background shapes define plasmid phenotypes: rectangle, multiple antibiotic resistance; oval, toluene/xylene degradation; circle, caprolactam degradation; no shape (clear background), naphthalene degradation. Bootstrap values (out of 1000 replicates) are shown adjacent to branch nodes. pBBR1 sequences were used to root the trees (pBBR1 branches are shortened for convenience). The lengths of horizontal branches correspond to evolutionary distances and the scale bars show the number of substitutions per site.

The IncP-9 plasmid phylogeny was inferred using sequences of both *rep* and *oriV*, in order to increase accuracy of the analysis. These regions were amplified from each IncP-9 plasmid (Table 1) using *rep*- and *oriV*-specific primers (Supplementary Table S1) that should yield products of 398 and 512–537 bp respectively. PCR products for *rep* and *oriV* were obtained with 23 and 25 IncP-9 plasmids, respectively (Table 1); only pBS240 and pBS243 gave neither *rep* nor *oriV* products. The PCR products were sequenced (*oriV* and *rep* sequences of pFKY1 were provided by M. Tsuda, Tohoku University) and multiple sequence alignments were created using UWGCG and clustal_x software (excluding the primer sequences). The *rep*–*oriV* sequence from *Bordetella bronchiseptica* plasmid pBBR1 (co-ordinates 1850–2635), which is distantly related to IncP-9 plasmids but compatible with pMT2 (Greated *et al.*, 2000), was used as an outgroup. The resulting neighbour-joining *rep* and *oriV* IncP-9 phylogenies (Fig. 1) revealed significant polymorphism within the family, with 30 plasmids placed in nine subgroups.

The subgroups were named with Greek letters (*α*, *β*, *γ*, *δ*, *ε*, *ŋ*, *ζ*, θ and ι subgroups; Fig. 1), in accordance with previous studies of IncP-9 plasmid diversity (Izmalkova *et al.*, 2006; Krasowiak *et al.*, 2002; Krasowiak, 2003; Leuchuk *et al.*, 2006). IncP-9 plasmids from different phylogenetic clusters showed 74–92 % (*rep*) and 64–93 % (*oriV*) DNA sequence identity, while the plasmids from the same subgroup shared 98–100 % identity for these determinants. Although subgroups tend to be associated with a single phenotypic profile, there are exceptions (e.g. the *β* cluster includes both naphthalene and toluene degradation). Apart from the additional ι cluster in the *oriV* tree (see below), the *rep* and *oriV* phylogenies have similar clusters, thus providing no evidence for incidents of recombination in the replication region. The results were not affected by the specific type of analysis used, since maximum-parsimony analysis also showed identical and well-supported phylogenetic clusters (data not shown), differing only slightly in their branching patterns.

Two Nah plasmids, pNL22 and pNL25, were allocated to a separate subgroup (ι) based on their *oriV* sequences, which showed only 64–67 % identity with other IncP-9 *oriV* sequences (Fig. 1). These plasmids gave no PCR products with other available IncP-9 replicon-specific primers (including those targeting *rep*), regardless of whether the DNA template used was total genomic DNA or purified plasmid DNA. Genomic DNA extracted from bacteria harbouring pNL25 yielded a very faint hybridization signal with the pMT2-derived *rep* probe (*α*), while the pNL25-derived *oriV* probe did not hybridize with plasmids from the *α*, *β* and *δ* subgroups under the stringency conditions used (data not shown). This suggests that the plasmids of the ι subgroup are highly divergent. In classical incompatibility tests with pNL22 and pNL25 plasmid-bearing bacteria, the naphthalene-degradation phenotype was lost with 100 % frequency upon the transfer of pM3 and R2 (Leuchuk *et al.*, 2006). If they possess a second, unrelated, replication and partitioning system they should not be displaced in this way, so pNL22 and pNL25 depend on a functionally related IncP-9 replication and/or partitioning system.

### Analysis of diversity in the common backbone of phylogenetically distant IncP-9 plasmids

**Fig. 2. f2:**
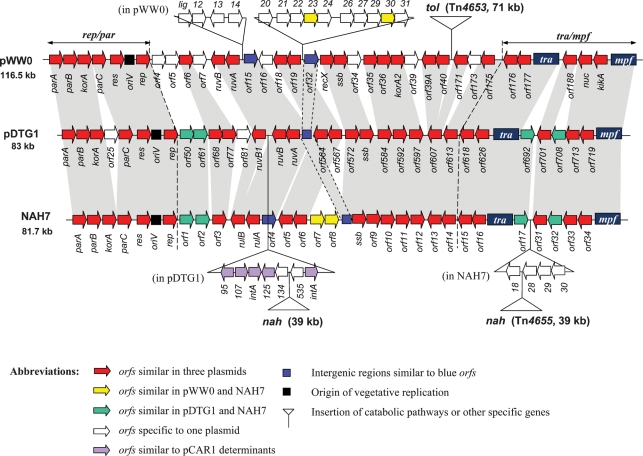
Schematic representation of alignment of pWW0, pDTG1 and NAH7 plasmid sequences generated using Artemis software. Grey areas connect similar *orfs*. The *tra* and *mpf* blocks included *traD–C* and *mpfJ–R* genes showing perfect synteny in three plasmids. Not to scale.

Genome-wide comparison of sequenced IncP-9 plasmids pWW0 (Greated *et al.*, 2002) and pDTG1 (Dennis & Zylstra, 2004) using the Artemis Comparison Tool (ACT) (Fig. 2) provided information about (i) the structure of the common IncP-9 plasmid backbone, (ii) the existence of possible unique regions in each plasmid, (iii) the levels of sequence identity for homologous segments and (iv) insertions of relevant degradation genes; this informationwas used to design tools for comparison of unsequenced plasmid genomes. Subsequent publication of the NAH7 sequence (Sota *et al.*, 2006) confirmed the identification of the ∼35 kb IncP-9 core (34.7, 35.0 and 34.1 kb in pWW0, pDTG1 and NAH7, respectively) including plasmid replication (*oriV*-*rep*) and stable maintenance (*par* and *res*) functions (4.4–4.9 kb), *tra*–*mpf* clusters encoding conjugative transfer apparatus (∼20 kb) and an additional 10 kb segment (variable region; Dennis & Zylstra, 2004). The list of the plasmid backbone determinants together with pair-wise DNA and protein sequence identities is given in Supplementary Tables S2 and S3. To extend our understanding of the sequence divergence in the variable region of the common IncP-9 backbone as well as of the possibility of recombination between related plasmids, PCR primers targeting selected conserved loci in pWW0 and pDTG1 were designed and corresponding pWW0-specific hybridization probes were produced (Fig. 2, Supplementary Table S1). Five primer pairs targeted homologous ORFs in pWW0 and pDTG1 (*orf6–7*, *ruvB*–*ruvA*, *orf18–19*, *orf39A–40* and *orf175* in pWW0, and their homologues *orf68–77*, *ruvB*–*ruvA*, *orf564–567* and *orf607–613* in pDTG1), while two were specific to pWW0 ORFs (*orf31–32* and *orf176–177*). Overall, 13 ORFs from pWW0 and eight from pDTG1 (primarily from the variable backbone segment) were covered by these primer pairs.

**Fig. 3. f3:**
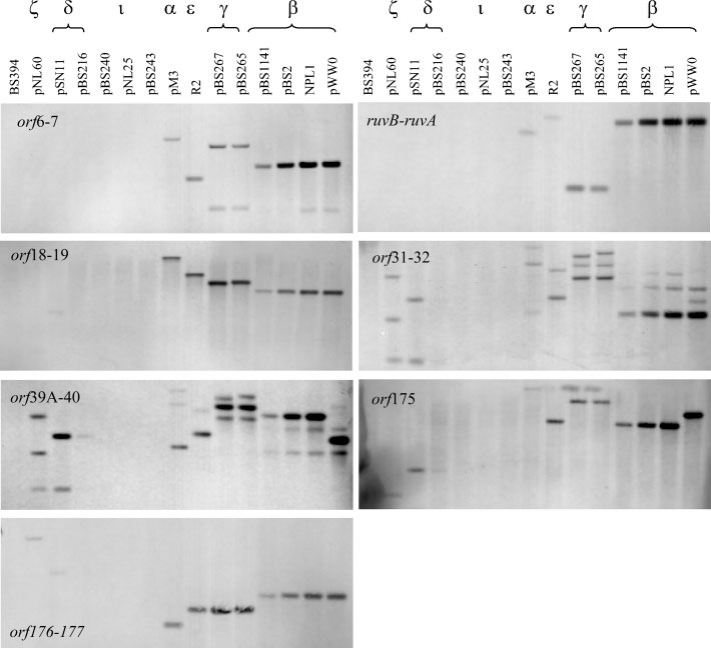
Hybridization of selected IncP-9 plasmids with pWW0-derived DIG-labelled probes specific to particular backbone loci. For hybridization, genomic DNA isolated from plasmid-containing bacteria was used; genomic DNA from plasmid-free *P. putida* BS 394 was used as a negative control (lane ‘BS394’). The DNA was digested with *Ava*I (*orf6–7* and *ruvB–ruvA* loci) or *Sal*I (others) before loading of the agarose gels, and was hybridized with pWW0-derived probes as described in Methods. In several hybridizations, multiple bands appeared in pWW0 and some test plasmids (*orf6*–*orf7*, *orf31*–*orf32*, *orf39A*–*orf40*). This might result from (i) incomplete digestion of the genomic DNA, (ii) non-specific hybridization or (iii) the presence of another region of homology elsewhere on the plasmid. The last could account for the appearance of multiple bands in the hybridizations of pWW0 and closely related *β* plasmids with *orf31*–*32* and *orf39A–40* probes, since pWW0*orf32* and pWW0*orf40* show 92.7 and 78.7 % nucleotide sequence identity to pWW0*orf15* and pWW0*orf171*, respectively (Fig. 2).

Twelve IncP-9 plasmids representing seven phylogenetic subgroups and the two unclassified plasmids pBS240 and pBS243 (Table 2) were analysed by PCR with these primer pairs and by Southern blotting of digested genomic DNA with hybridization probes. A PCR product of the expected size (∼500 bp) or a visible hybridization signal were considered to be positive results. The results of the PCR and hybridization experiments are summarized in Table 2 and representative hybridization blots are shown in Fig. 3. The results showed that all backbone loci studied were detected in nearly all IncP-9 plasmids tested by either PCR or hybridization analysis or both. Where only one method gave a positive result this might indicate local variations in the level of sequence conservation. The results indicate that the proposed backbone is indeed present in the majority of IncP-9 plasmids. Three plasmids, pNL25 (ι), pBS240 and pBS243 [classified as IncP-9 plasmids on the basis of incompatibility testing (I. A. Kosheleva, unpublished data)], did not yield PCR products or hybridization signals with any primers or probes targeting the backbone determinants. This suggests that these plasmids have a higher level of sequence divergence in the backbone functions. As expected, PCR with pWW0- or pDTG1-specific primers yielded products primarily with plasmids from their cognate subgroups. Plasmid R2 (ε) gave PCR products with all but one pWW0-specific primer, while pM3 (*α*) and pBS265/267 (*γ*) gave PCR products with only two and one primer pairs, respectively, while none of the plasmids from pWW0 branch (*β*, *α*, ε, *γ* subgroups) worked with pDTG1-specific primers. However, they all yielded hybridization signals with pWW0-derived probes, consistent with them being more closely related to pWW0 than to pDTG1 (Fig. 1). Plasmids from *δ* (pBS216 and pSN11) and *ζ* (pNL60) subgroups gave PCR products with all or almost all of the pDTG1-specific primers. In contrast, they worked with only one pWW0-specific primer pair (*orf176–177*) and often yielded weak signals in hybridizations with pWW0 probes (Fig. 3). Peculiarly, *orf6–7* and *ruvB*–*ruvA* were not detected in the *δ* and *ζ* plasmids by hybridization with pWW0-specific probes, while PCR with equivalent pDTG1-specific primers gave products of the expected sizes (*orf68*–*77* and *ruvA*–*ruvB* pairs in pDTG1). This could be due to high sequence divergence in these loci between pWW0 and the plasmids from the *δ* and *ζ* subgroups. The DNA sequence identity for pWW0 *orf6–7* and *ruvB*–*ruvA* and their homologues in pDTG1 (*orf68–7* and *ruvA*–*ruvB*, respectively) ranged from 51.3 to 68.7 %, while it was 69.4–78.2 % for other backbone loci analysed (Supplementary Table S2).

The data were also scanned for abnormal patterns of hybridization that were not typical of the plasmid classification by *ori–rep* phylogeny. The PCR results (Table 2) showed that for only one plasmid from the pWW0 branch, R2 (ε), were products amplified with the pWW0-specific primers as well as they were with the *β* plasmid pBS2 (both plasmids only failed to yield products with *orf39A–40* primers). This indicates that R2 might share significant sequence similarity with the backbone of both pWW0 and the *β* plasmids, although their *rep* and *oriV* sequences are as dissimilar (12–13 % divergence) as, for instance, those of pWW0 and *α* or *γ* plasmids (12–15 %). Plasmid pSN11 (*δ*) gave a very strong hybridization signal with the pWW0 *orf39A–40* probe, which also may suggest recombination in this region. These cases should be investigated further by additional PCR/hybridization and sequencing analysis to test whether these results do indeed arise from mosaics created by recombination between conserved IncP-9 backbone regions in diverse members of the family.

### Analysis of possible insertions of accessory DNA in diverse IncP-9 plasmids

**Fig. 5. f5:**
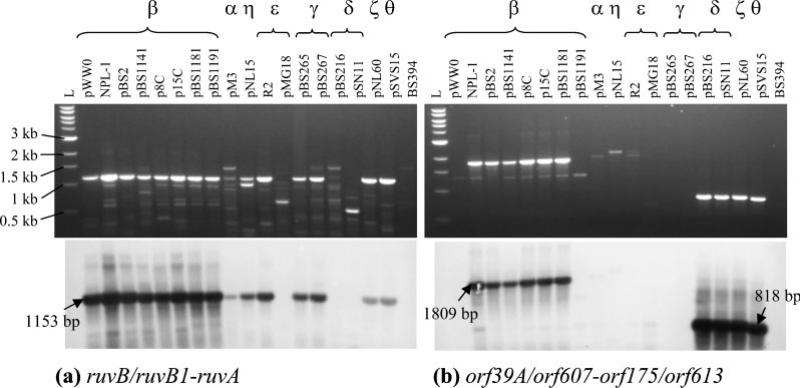
PCR and hybridization analyses of selected IncP-9 plasmids with the primers and hybridization probes targeting the sites of accessory DNA insertions in the common plasmid backbone. (a) PCR products obtained from IncP-9 plasmids with the *ruvB*-F(2)–*ruvA*-R(2) primers and subsequently hybridized with the pWW0-derived *ruvB*/*ruvB1*-*ruvA* DIG-labelled hybridization probe. (b) PCR products obtained with the 39A/607F–175/613R primers and hybridized with the pBS216-derived hybridization probe *orf39A*/*orf607*–*orf175*/*orf613*. PCR with genomic DNA from the plasmid-free *P. putida* BS 394 was used as a negative control (lane ‘BS394’).

PCR and hybridization analyses were used to determine whether diverse IncP-9 plasmids have insertions of accessory DNA (degradation and resistance genes) in the same backbone sites as in those of pWW0 and pDTG1. To target these sites, the PCR primers 39A/607F–175/613R and ruvB-F(2)–ruvA-R(2) were designed on the basis of the conserved sequences in the determinants surrounding the catabolic gene cassette in one plasmid and in their homologues in other plasmids (Figs 2 and 4). Thus, *orf39A* and *orf175* flank Tn*4653* carrying the toluene-degradation pathway in pWW0, while the equivalent ORFs in pDTG1 were *orf607* and *orf613*. Similarly, another primer pair was designed in the *ruvB*–*ruvA* (*ruvB1*–*ruvA* in pDTG1) region, since in pDTG1 the *nah* gene insertion occurred in *ruvB*. As expected pWW0 and pDTG1 gave PCR products with only one primer pair, whose sizes should be 1153 and 818 bp, respectively. PCR products obtained from different IncP-9 plasmids were analysed on agarose gels and hybridized with probes derived from pWW0 (for the *ruvB*-F(2)–*ruvA*-R(2) amplicons) or from pBS216 (similar to pDTG1) (for the 39A/607F–175/613R amplicons), as described in Methods. The results are summarized in Table 3 and Fig. 5.

Results with primers and probes specific to the *ruvB*–*ruvA* locus (Fig. 5a[Fig f5]) indicated that this region was intact and of the same length (1.2 kb) in all of the plasmids used, apart from the *δ* plasmids (pBS216, pSN11) and pMG18 (ε). Therefore, only plasmids from the *δ* subgroup and possibly pMG18 might carry insertions in *ruvB*. The PCR and hybridization analysis of the *orf39A*/*607*–*orf175*/*613* locus (Fig. 5b[Fig f5], Table 3) indicated that it was not intact in the following plasmids: pWW0 (*β*, contains transposon insertion in this region), pBS1191 (*β*), pM3 (*α*), pNL15 (*ŋ*), pBS265 and pBS267 (*γ*), and R2 and pMG18 (*ε*). Interestingly, the majority of Nah plasmids of the *β* subgroup (NPL-1, pBS2, p8C, p15C, pBS1141 and pBS1181) yielded a PCR product of ∼1.8 kb. This is very close to the size of an intact *orf39A–orf175* region in pWW0 (1809 bp) if it lacked the insertion of Tn*4653*. These results indicate that Nah plasmids from the *β* subgroup may be very similar to pWW0 and may carry their accessory DNA in the same position (i.e. between *orf39A* and *orf175*; plasmid pBS1191) as well as different backbone sites, but not in *ruvB* as in pDTG1 (plasmids NPL-1, pBS2, p8C, p15C, pBS1141 and pBS1181). Plasmids from the *α*, *ŋ*, *γ* and ε clusters may also contain insertions in the same region as pWW0, while in pMG18 (*ε*) both backbone sites analysed might be disrupted by insertions. The pDTG1-related plasmids (*δ*, *ζ* and θ) yielded an approximately 800 bp PCR product, indicating the integrity of the *orf39A*/*607*–*orf175*/*613* backbone site. Sequencing of NAH7 (*ζ* subgroup) confirmed that in this plasmid the *nah* cluster was inserted in a different, third, backbone location, between the *tra* and *mpf* genes (Sota *et al.*, 2006), consistent with the results obtained for our *ζ* plasmid (pNL60).

## DISCUSSION

From 30 IncP-9 plasmids this study distinguished nine IncP-9 subgroups (*α* to ι) based on divergence in *rep* and *oriV* sequences. The results are consistent with the existing molecular classifications of IncP-9 plasmids (Izmalkova *et al.*, 2006; Krasowiak *et al.*, 2002; Krasowiak, 2003; Leuchuk *et al.*, 2006) but provide much more reliable distances between the different subgroups as well as confirming the existence of the two main clusters of subgroups. Previous RFLP and sequence analysis of PCR products amplified from replication/maintenance loci in a small selection of IncP-9 plasmids has identified four plasmid subgroups, *α*, *β*, *γ* and ε (Krasowiak *et al.*, 2002; Krasowiak, 2003). The *δ* subgroup identified by Izmalkova *et al.* (2006) included plasmids that were positive only in PCR with the IncP-9 *rep* primers (Greated & Thomas, 1999) but not with other available IncP-9-specific primers (Krasowiak *et al.*, 2002). Finally, several Nah plasmids were allocated to ** and *ζ* subgroups by RFLP analysis of their *rep* products, and to the IncP-9-like replicons (ι subgroup) by incompatibility testing (Leuchuk *et al.*, 2006).

The *oriV* and *rep* phylogenies were rooted using replication region sequences from the pBBR1 plasmid (Antoine & Locht, 1992). This showed two main divergent lineages in the evolution of IncP-9 plasmid family: the ‘pWW0 branch’, including clades *α*, *β*, *γ*, *ε* and *ŋ*, and the ‘pDTG1 branch’, comprising the *δ*, *ζ* and θ subgroups (Fig. 1). Nucleotide sequence divergence between plasmids from these branches was highest (22–26 % for *rep* and 24–28 % for *oriV*), while that between subgroups of each branch ranged from 7 to 19 %. Consistent with this, only plasmids from the pWW0-branch (*α*, *β*, *γ*, ε and ** clusters) but not those from the pDTG1-branch (*δ*, *ζ* and θ cluster) yielded PCR products with primers based on replication and maintenance loci of pMT2 (*α*) (Izmalkova *et al.*, 2006; Krasowiak *et al.*, 2002; Y. R. Sevastsyanovich and others, unpublished results). Our PCR and hybridization analyses and the sequence analysis of NAH7 (Sota *et al.*, 2006) also support the division of the IncP-9 plasmid family into two major clusters (pWW0 and pDTG1 branches).

Three subgroups (*α*, ε and *ŋ*) included multiple antibiotic-resistance plasmids isolated from clinical and environmental samples. Diverse plasmids encoding degradative functions (for naphthalene, toluene/xylenes and caprolactam) formed the six remaining subgroups, each including plasmids of the same phenotype, with the exception of the *β* branch, which included Tol and Nah plasmids. Several IncP-9 plasmids that did not give PCR products with the IncP-9 replicon-specific primers used may create additional subgroups, characterized by higher sequence divergence in the replication region.

The high sequence variability within the replication region of IncP-9 plasmids contrasts, for example, with that exhibited by the IncP-4 (IncQ) and IncP-1 plasmid families. The IncP-4 family is currently divided into three subgroups on the basis of diversity in the replication initiator RepC (Rawlings & Tietze, 2001; Rawlings, 2005). The IncP-1 group includes five subgroups, as demonstrated by the phylogenetic analysis of the determinants involved in replication, maintenance, global gene regulation and conjugal transfer (Bahl *et al.*, 2007; Haines *et al.*, 2006; Harada *et al.*, 2006; Heuer *et al.*, 2004; Vedler *et al.*, 2004). However, the DNA sequence divergence in the replication initiator gene from the plasmids allocated to different IncP-1 subgroups was 22–29 %, with up to 16 % divergence in the *β* branch alone. On this basis, some of the IncP-9 subgroups might be considered as a single group, since the divergence between them was only 7 %. On the other hand, the variation among the IncP-1*β* plasmids was relatively continuous, while the IncP-9 plasmids fall more tightly into the groups we have defined, suggesting more distinct clades.

Comparison of the complete sequences of pWW0, pDTG1 and NAH7 identified a 35 kb core composed of a 5 kb replication/stability gene cluster, a 20 kb conjugal transfer/mating pair formation cluster and an additional 10 kb variable region, which probably does not encode functions essential for plasmid survival (Dennis & Zylstra, 2004; Sota *et al.*, 2006). Apart from the atypical IncP-9 replicons (ι subgroup and pBS240 and pBS243), the diverse plasmids analysed here possess a variable backbone segment (10 kb), similar to either the pWW0 or the pDTG1 type, suggesting conservation throughout the IncP-9 family. A blast search of GenBank identified pBI709 (partial sequence accession no. AY299015) from *Pseudomonas* as an additional family member based on extensive sequence similarity with the IncP-9 backbone (65–72 % overall DNA sequence identity and 52–81 % protein sequence identity between pBI709 and sequenced IncP-9 plasmids; Supplementary Table S3).

Recent studies have suggested that recombination between related plasmids might be more common than previously assumed for incompatible replicons. For example, F-related plasmids of the *E. coli* reference collection (ECOR) carry recombinant *repA*, *finO* and *traD* genes, as detected by hybridization analysis (Boyd *et al.*, 1996). Sequencing of plasmid pB10 has revealed that its stable inheritance module (*klcAB*–*korC* and *kleAEF*) is closely related to that from pB4, while the rest of the backbone is more similar to that of R751, pTSA and pADP-1 (all plasmids belong to the IncP-1*β* subgroup; Schluter *et al.*, 2003). The hybridization experiments presented in this study indicated that some plasmids might be the product of recombination between diverse IncP-9 replicons. For example, R2 has an ε plasmid replication region but shares extensive similarity with *β* (pWW0) plasmids in the variable backbone segment. In addition, R2 may carry its accessory DNA in the same backbone site as in pWW0. The plasmids that showed unexpectedly strong or weak hybridization with the pWW0 sequences [e.g. pSN11, pBS216 (*δ*) and pBS1141 (*β*)] may be recombinants of the corresponding backbone loci. More extensive hybridization and sequence analysis involving other parts of the backbone would be worthwhile in order to address the possibility of recombination in this plasmid group.

In the sequenced IncP-9 plasmids three different sites for integration of accessory DNA were identified: at the junction between the two modules, *tra* and *mpf* (NAH7), as in the IncP-1 plasmids; in pDTG1*ruvB*; and between pWW0*orf40* and pWW0*orf171*. The results of this study indicated that diverse IncP-9 replicons carry the insertions of the genes encoding adaptive traits in these, as well as additional, backbone sites. This is consistent with the notion that similar or identical degradative markers have been acquired multiple times by IncP-9 plasmids, raising the question of whether IncP-9 plasmids possess specific features that make them particularly suited to carry such determinants through inherent plasmid properties such as host range, copy number or even effects on host structure or physiology.

## Figures and Tables

**Fig. 4. f4:**
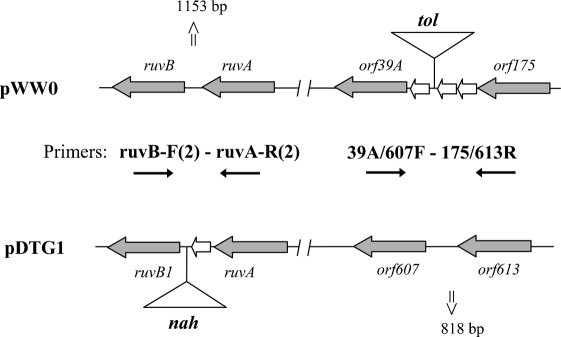
Schematic representation of the pWW0 versus pDTG1 plasmid alignment to show the location of the conserved primers specific to determinants flanking the insertions of corresponding catabolic pathways. The conserved primer pairs are shown by black arrows; the genetic determinants used for the primer design are shown by grey arrows; white triangles (*tol* and *nah*) indicate insertions of toluene and naphthalene catabolic pathways, respectively. The sizes of the expected PCR products are indicated.

**Table 1. t1:** Strains used in this study Cb, Gm, Km, Sm, Su, Tc and Hg, resistance to antibiotics carbenicillin, gentamicin, kanamycin, streptomycin, sulphonamide, tetracycline and to mercury ions, respectively; Uv, ultraviolet light protection; Ant, Cap, Nah, Phn, Sal, Tol and Xyl, ability to degrade anthracene, ε-caprolactam, naphthalene, phenanthrene, salicylic acid, toluene and (*m*- and *p*-) xylenes, respectively.

**Strain**	**Plasmid**	**Size (k)b**	**Plasmid phenotype**	**Source of isolate**	Reference or provider*
*P. putida* PpG7	NAH7	83	Nah Sal	Coal-tar-contaminated soil, CA, USA	Dunn & Gunsalus (1973)
*P. putida* BS 202	NPL-1	100	Nah Sal	Coal-tar-contaminated soil near coal tar mine, Makeevka, Ukraine	Izmalkova *et al.* (2006); RAS
*Pseudomonas* spp. 8C	p8C	110	Nah Sal Phn	Oil-contaminated soil, Tumen region, Western Siberia, Russia	Izmalkova *et al.* (2006); RAS
*Pseudomonas* spp. 15C	p15C	110	Nah Sal Phn	Oil-contaminated soil, Tumen region, Western Siberia, Russia	Izmalkova *et al.* (2006); RAS
*P. putida* BS 238	pBS2	130	Nah Sal	Soil from territory of metallurgical plant, Nizhniy Tagil, Russia	Izmalkova *et al.* (2006); RAS
*P. putida* BS 3710 (*cys^−^*)	pBS216	83	Nah Sal Phn	Soil from territory of metallurgical plant, Magnitogorsk, Russia	Izmalkova *et al.* (2006); RAS
*P. putida* BS 639 (*cys^−^*)	pBS240	160	Nah	Coke chemical plant, Kemerovo, Russia	RAS
*P. putida* BS 638 (*cys^−^*)	pBS243	160	Nah	Soil from territory of metallurgical plant, Magnitogorsk, Russia	RAS
*P. putida* BS 394 (*cys^−^*)	pBS265	130	Cap	Chemical plant sewage, Severodonetsk, Ukraine	Krasowiak *et al.* (2002); RAS
*P. putida* BS 394 (*cys^−^*)	pBS267	130	Cap	Chemical plant sewage, Severodonetsk, Ukraine	Krasowiak *et al.* (2002); RAS
*P. putida* BS 394 (*cys^−^*)	pBS268	85	Cap	Chemical plant sewage, Kemerovo, Russia	Mavrodi *et al.* (2003); RAS
*P. putida* BS 3701	pBS1141, pBS1142	100, 60	Nah Sal Phn Ant cryptic	Coke chemical plant, Vidnoe, Moscow region, Russia	Izmalkova *et al.* (2006); RAS
*P. putida* BS 3750	pBS1181	120	Nah Sal Phn	Oil-contaminated soil, Tumen region, Western Siberia, Russia	Izmalkova *et al.* (2006); RAS
*P. putida* BS 3790	pBS1191, pBS1192	100, 60	Nah Sal Phn Ant cryptic	Oil-contaminated soil, Tumen region, Western Siberia, Russia	Izmalkova *et al.* (2006); RAS
*P. putida* NCIB 9816-4	pDTG1	83	Nah	Coal-tar-contaminated site, Bangor, Wales, UK	Dennis & Zylstra (2004); Evans *et al.* (1965)
Unknown†	pFKY1	200	Nah Sal	Oil-contaminated site, Japan	M. Tsuda, unpublished data
*P. putida* M	pM3	75	Sm Tc Uv	Sewage and soil from different industrial and agricultural locations in Belarus and Azerbaijan	Greated *et al.* (2000); Titok *et al.* (1991)
*P. putida* M (*pro^−^*)	pM77	75	Sm Tc	Soil from the area of sewage treatment plant, Minsk, Belarus	Krasowiak *et al.* (2002); BSU
*P. putida* M (*pro^−^*)	pM80	75	Sm Tc	Soil from the area of sewage treatment plant, Minsk, Belarus	Krasowiak *et al.* (2002); BSU
*P. putida* AC34	pMG18	100	Cb Gm Km Sm Su Hg	Japan	Jacoby & Matthew (1979)
*P. putida* 10a	pNL4	75	Nah Sal	Soil from a distillery area, Minsk, Belarus	Leuchuk *et al.* (2006); BSU
*E. coli* C600‡	pNL15‡	75	Sm	Soil from a petrol station area, Minsk, Belarus	Leuchuk *et al.* (2006); BSU
*Pseudomonas fluorescens* 41a	pNL22	100	Nah Sal	Soil from a petrol station area, Minsk, Belarus	Leuchuk *et al.* (2006); BSU
*P. putida* 21a	pNL25	75	Nah Sal	Soil from a railway station area, Minsk, Belarus	Leuchuk *et al.* (2006); BSU
*Pseudomonas* spp. 58	pNL29	nd§	Nah Sal	Soil from a petrol station area, Minsk, Belarus	Leuchuk *et al.* (2006); BSU
*Pseudomonas aeruginosa* 56	pNL31	nd§	Nah Sal	Soil from the roadside, Minsk, Belarus	Leuchuk *et al.* (2006); BSU
*P. fluorescens* 18d	pNL60	120	Nah Sal	Soil from the foundry area, Homel, Belarus	Leuchuk *et al.* (2006); BSU
*Pseudomonas aureofaciens* OV17	pOV17	85	Nah	Oat rhizosphere from oil-contaminated soil, Western Siberia, Russia	RAS
*P. putida* SN11	pSN11	83	Nah Sal	Salt-contaminated soil from chemical plant, Berezniki, Ural, Russia	Izmalkova *et al.* (2006); RAS
*P. putida* SVS15	pSVS15	90	Tol Xyl	Piece of rubber from used-car-tyre storage, Minsk, Belarus	Sentchilo *et al.* (2000)
*P. putida* mt-2 (PaW1)	pWW0	116.58	Tol Xyl	USA	Greated *et al.* (2002); Williams & Murray (1974)
*P. aeruginosa* ML 4262	R2	73	Cb Sm Su Uv	Japan	Kawakami *et al.* (1972)

*RAS and BSU, bacterial strain collections obtained from Russian Academy of Sciences and Belarus State University, respectively. †Plasmid obtained by exogenous isolation. ‡Plasmid was labelled with mini-Tn*5* (Km) and transferred by conjugation into *E. coli* C600 (selection for Km Nah^−^ phenotype) from the natural host *P. fluorescens* 42 (Leuchuk *et al.*, 2006). §Not determined.

**Table 2. t2:**
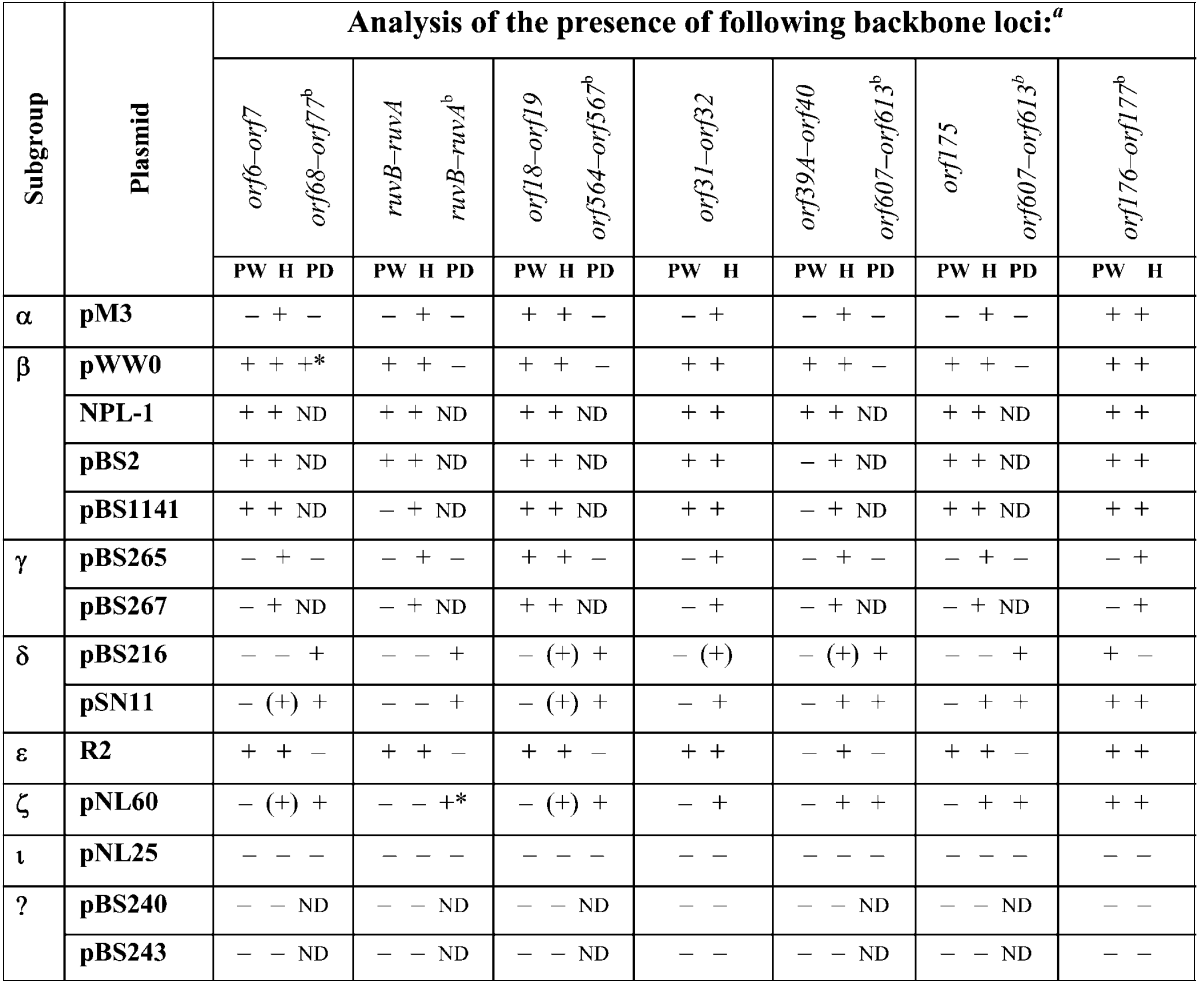
PCR- and hybridization-based detection of the selected backbone loci in plasmids representing seven of the nine IncP-9 subgroups using pWW0- and pDTG1-specific tools

*a*, +, (+) and − correspond to strong, weak and no PCR or hybridization signal, respectively. PW, PCR with pWW0-specific primers; H, hybridization with pWW0-specific probes; PD, PCR with pDTG1-specific primers. An asterisk indicates a PCR product larger than the expected size. nd, Not determined. *b*, Pairs of homologous backbone loci from pWW0 and pDTG1 are indicated in this order; if only one *orf* pair is indicated, it belongs to pWW0.

**Table 3. t3:** Analysis of possible insertions in two sites of the IncP-9 backbone by PCR and hybridization with plasmids representing the eight best-characterized subgroups

**Subgroup**	Plasmid phenotype*	**Plasmid**	***ruvB*/*ruvB1*–*ruvA***		***orf39A*/*607*–*orf175*/*613***	
			**Hybridization signal**	**Presence of insertion†**	**Hybridization signal**	**Presence of insertion†**
*α*	R	pM3	+	−	−	+
*β*	Tol	pWW0	+	(−)	−	(+)
	Nah	NPL-1, pBS2, pBS1141, p8C, p15C, pBS1181	+	−	+	−
	Nah	pBS1191	+	−	−	+
*γ*	Cap	pBS265, pBS267	+	−	−	+
*δ*	Nah	pBS216, pSN11	−	(+)	+	(−)
ε	R	R2	+	−	−	+
	R	pMG18	−	+	−	+
θ	Tol	pSVS15	+	−	+	−
*ŋ*	R	pNL15	+	−	−	+
*ζ*	Nah	pNL60	+	(−)	+	(−)

*Tol, Nah and Cap, degradation of toluene/xylenes, naphthalene and caprolactam, respectively; R, antibiotic resistance. †+ or − indicates predicted presence or absence of insertions in uncharacterized plasmids. (+) or (−) indicates known presence or absence of insertions in characterized plasmids (pWW0, *δ* and *ζ* plasmids).

## Abbreviations

- HGT, horizontal gene transfer
